# A Self‐Conditioned Metalloporphyrin as a Highly Stable Cathode for Fast Rechargeable Magnesium Batteries

**DOI:** 10.1002/cssc.202100340

**Published:** 2021-03-16

**Authors:** Ebrahim Abouzari‐Lotf, Raheleh Azmi, Zhenyou Li, Shirin Shakouri, Zhi Chen, Zhirong Zhao‐Karger, Svetlana Klyatskaya, Julia Maibach, Mario Ruben, Maximilian Fichtner

**Affiliations:** ^1^ Electrochemical Energy Storage Helmholtz Institute Ulm (HIU) Helmholtzstraße 11 89081 Ulm Germany; ^2^ Institute of Nanotechnology and Institute of Quantum Materials and Technology Karlsruhe Institute of Technology P.O. Box 3640 76021 Karlsruhe Germany; ^3^ Institute for Applied Materials-Energy Storage Systems Karlsruhe Institute of Technology 76344 Eggenstein-Leopoldshafen Germany; ^4^ Institute of Quantum Materials and Technology Karlsruhe Institute of Technology P.O. Box 3640 76021 Karlsruhe Germany; ^5^ Centre Européen de Science Quantique (CESQ) Institut de Science et d'Ingénierie Supramoléculaires (ISIS) Université de Strasbourg 8, Allée Gaspard Monge 67000 Strasbourg France

**Keywords:** electrode materials, magnesium batteries, multi-electron redox reactions, porphyrins, transmetalation

## Abstract

Development of practical rechargeable Mg batteries (RMBs) is impeded by their limited cycle life and rate performance of cathodes. As demonstrated herein, a copper‐porphyrin with meso‐functionalized ethynyl groups is capable of reversible two‐ and four‐electron storage at an extremely fast rate (tested up to 53 C). The reversible four‐electron redox process with cationic‐anionic contributions resulted in a specific discharge capacity of 155 mAh g^−1^ at the high current density of 1000 mA g^−1^. Even at 4000 mA g^−1^, it still delivered >70 mAh g^−1^ after 500 cycles, corresponding to an energy density of >92 Wh kg^−1^ at a high power of >5100 W kg^−1^. The ability to provide such high‐rate performance and long‐life opens the way to the development of practical cathodes for multivalent metal batteries.

## Graphical abstract

**Self‐conditioning**: The metalloporphyrin based electrodes exhibit reversible insertion/deinsertion of Mg^2+^ and bulky anions as well as excellent capacity retention over several hundred cycles at very fast charge‐discharge rates. This opens the pathway for development of new organic electrode materials for stable high‐energy and high‐power multivalent metal‐based energy storage systems.

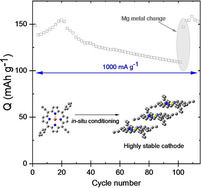

Lithium‐ion‐based electrochemical energy storage systems are still the main choice in the power battery market with proven versatility across a broad range of application requirements from portable electronics to electric vehicles and grid applications.^[1]^ While significant improvements have been achieved recently in the chemistry of the working electrodes,^[2]^ low relative abundance of materials (e. g., lithium and cobalt) and the high associated energy cost (considering the production of materials, batteries, and recycling) are still challenging.^[3]^ Such facts strongly demand to move towards post‐Li chemistries including abundant and cheap monovalent (Na, K) and divalent (Mg, Ca) metals.^[4]^ Rechargeable Mg batteries (RMBs) utilizing a Mg anode are promising candidates for applications beyond Li‐ion batteries owing to the Mg abundance (around 10^4^ times more than that of Li in the earth‘s crust) and its bivalent nature (theoretically facilitates higher volumetric charge capacity compared to the monovalent Li‐ and Na‐ion batteries).^[5]^ Additionally, Mg is less prone to forming metallic dendrites in comparison with the Li metal, and therefore the respective RMBs would be safer.

Tremendous advances in electrolyte development have been achieved in the past years, resulting in practical and non‐corrosive magnesium electrochemistry.[^6^, ^7^] Nevertheless, the development of high‐performance cathode materials has so far limited the commercial viability of RMB technology.[^8^, ^9^, ^10^, ^11^, ^12^, ^13^] In fact, the bivalent nature of the magnesium ion results in strong interactions with insertion electrode materials, and consequently, the diffusion of ions in the materials can be orders of magnitude more sluggish than that of monovalent cations (e. g., Na and Li). In conventional intercalation cathodes, such kinetically sluggish Mg insertion/extraction results in low capacity and high voltage hysteresis as well as low energy density of RMBs.^[14]^ Moreover, long cycle life is still elusive in the high‐capacity conversion‐type cathodes (e. g., sulfur) owing to the loss of active material upon dissolution of polysulfides into the electrolyte during the sulfur redox process.^[15]^

In contrast to their inorganic counterparts, organic cathodes (e. g., quinone‐based materials) offer less rigid migration pathways for Mg^2+^ ions with lower migration barriers, which allow operation at higher rates. However, despite such advantages and the potential tuneability of redox properties in organic cathodes, the stable discharge capacity is usually quite below the theoretical values and the rate capability is limited.[^16^, ^17^, ^18^] Therefore, the development of cathode materials with high capacity and cycle life in RMBs remains challenging.

Facile reduction and oxidation of fused‐ring organics is well known, and metal complexes of porphyrin and phthalocyanine with promising redox behavior in monovalent metal‐ion‐based electrochemical energy storage systems of Li[^19^, ^20^] and K^[21]^ were reported recently. Introducing terminal alkyne functionality and a copper(II) cation as central atom was found to significantly improve the stability of [5,15‐bis‐(ethynyl)‐10,20‐diphenylporphinato]copper(II) (CuDEPP) upon cycling. In a first study, more than 60 % of the capacity was retained after 8000 cycles at a high current density of 4 A g^−1^ when CuDEPP was combined with a lithium negative electrode. A rapid redox reaction involving four‐electron transfer led to the gravimetric energy and power densities of as high as 345 Wh kg^−1^ and 29 kW kg^−1^, respectively. Encouraged by such remarkable results, we extended our study to explore the potential of CuDEPP as a universal cathode and in particular in a bivalent Mg‐ion based energy storage system. The small gap between the highest occupied molecular orbital (HOMO) and the lowest unoccupied molecular orbital (LUMO) of CuDEPP (Figure S1) is expected to improve the kinetics of interaction of the cathode with divalent ions. Moreover, the porous nature and high surface area of the synthesized CuDEPP (Figure S2a) are supposed to ease the accommodation of the large ions and facilitate the ion transport at the electrode/electrolyte interface, which bring about high rate performance and power density.^[22]^

Theoretically, the CuDEPP molecule can store four charges in reversible redox reactions including two‐electron reduction and two‐electron oxidation (Figure 1a) and amounting to a capacity value of 187 mAh g^−1^. While the Mg cation storage could be achieved at a low potential through the two‐electron reduction of metalloporphyrin, the two‐electron oxidation could be approached at higher potential and through anion charge compensation. A key requirement for a practical storage system utilizing CuDEPP is the desired electrolyte compatible with Mg anode, having good interfacial compatibility with the cathode and large electrochemical window. In fact, the majority of the Mg electrolyte components are prone to oxidation and highly corrosive, which practically limits the four‐electron storage in porphyrins.

**Figure 1:**
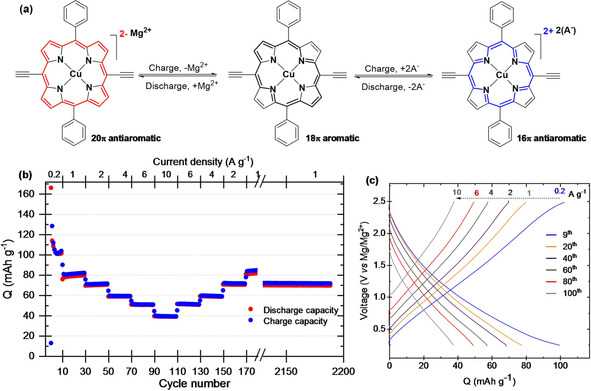
(a) General pathway of the two‐electron oxidation and two‐electron reduction reactions of CuDEPP involving reversible aromaticity switching. (b) Rate capability at various current densities from 0.2 to 10 A g^−1^ and (c) selected charge‐discharge curves of CuDEPP with a Mg(HMDS)_2_/MgCl_2_/PP_14_TFSI/THF electrolyte with total Mg^2+^ concentration of 0.625 M in the potential range of 0.2–2.5 V.

The synthesis of the abovementioned porous CuDEPP with large micron‐sized crystals is reported elsewhere.^[19]^ The as‐synthesized CuDEPP powder was ball‐milled to increase the surface area to 229.5 m^2^ g^−1^ (Figure S2b), whereby the particle size was reduced to the submicron scale (Figure S3). The electrochemical behavior of CuDEPP in RMB was evaluated in chloride‐based as well as in chloride‐free electrolytes (see Experimental in the Supporting Information). Utilizing Mg(HMDS)_2_/MgCl_2_/PP_14_TFSI (HMDS: hexamethyldisilazide; PP14TFSI: 1‐butyl‐1‐methylpiperidinium bis(trifluoromethylsulfonyl)imide) electrolyte, the CuDEPP cathode delivered a stable discharge capacity of 108 mAh g^−1^ with a coulombic efficiency (CE) of above 97 % after the third cycle at the charge‐discharge current of 200 mA g^−1^ (Figure 1b). Considering the capacity contribution from inactive electrode components (Figure S4b), the discharge capacity is proximate to the theoretical value of 93.5 mAh g^−1^ associated with the two‐electron redox reaction. The lack of a horizontal voltage plateau in the charge‐discharge profiles (Figure 1c) signifies rapid pseudo‐capacitive redox reactions. Besides, the limited capacity fade in the rate performance (Figure 1b) reflects the fast charge transfer kinetics of Mg^2+^ ions in the CuDEPP and/or within the electrolyte. Typically, the electrode delivered a capacity of 58 mAh g^−1^ within 52 s with a CE of 99.2 % when cycling at 21 C (4 A g^−1^). Remarkably, CuDEPP demonstrated excellent cycling stability and delivered an energy density of 75 Wh kg^−1^ (based on the active material) after 2000 cycles, which corresponds to a power density of 1055 W kg^−1^ and compares well with the benchmark organic cathode materials in RMBs (Table S1). However, there is an argument about the insertion chemistry of cations in chloride‐containing electrolytes, and, as shown recently, the storage of MgCl^+^ is dominant and the hybrid storage significantly reduces the cell energy.[^23^, ^24^, ^25^]

To explore the four‐electron redox potential of CuDEPP, an electrolyte with a larger electrochemical stability window is required. Herein, a Cl^−^ free, non‐corrosive electrolyte of magnesium tetrakis(hexafluoroisopropyloxy)borate (Mg[B(hfip)_4_]_2_) in dimethoxyethane (DME) solvent was used owing to the reported fast and efficient Mg deposition/dissolution as well as excellent oxidative stability of as high as 4.5 V against stainless steel (SS).^[10]^ After initial conditioning cycles, the CuDEPP cathode reveals stable discharge capacities and good rate capabilities. Figure 2a shows the rate capability at the current range of 0.2–10 A g^−1^. At a lower current density of 0.2 A g^−1^ (≈1 C), the discharge capacity is significantly higher than the theoretical value for a two‐electron based system. Notably, the cell could deliver the discharge capacity of 102 and 49 mAh g^−1^ when the current density increased to 1 and 10 A g^−1^ (≈5 and 53 C), respectively.

**Figure 2:**
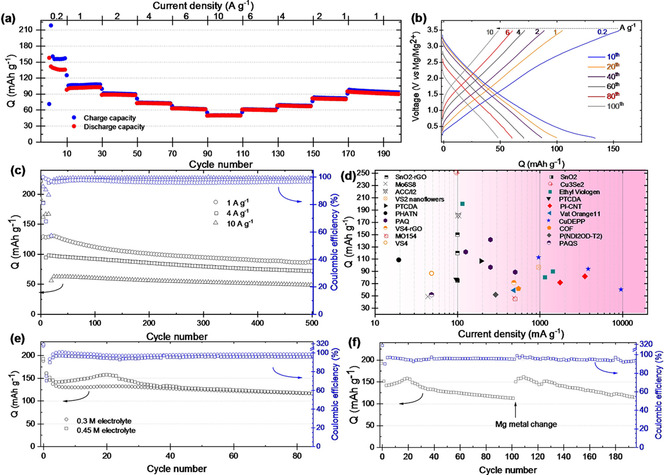
Electrochemical performance of the Mg/Mg[B(hfip)_4_]_2_ (0.3 M)/CuDEPP cell. (a) Rate capability with an increase in the charge‐discharge rate from 200 mA g^−1^ to 10 A g^−1^ and then a decrease to 1 A g^−1^. (b) Corresponding charge‐discharge curves of different cycles. (c) Cycling stability at the various current densities. (d) Stabilized capacity (after 100 cycles) of CuDEPP versus some other organic, inorganic, and polymeric cathodes for RMBs at various current densities (details in the Supporting Information; Table S2). Cycling performance of the CuDEPP at 1 A g^−1^ (e) with varied electrolyte concentration and (f) rebuilt cell after 100 cycles with a fresh Mg anode and renewed electrolyte.

Moreover, after the rate test of 150 cycles, the discharge capacity returned to 94 mAh g^−1^ at the current density of 1 A g^−1^, signifying a superior capacity recovery. The excellent rate performance and good capacity contribute to the high energy and power densities. The CuDEPP can deliver an energy density of 125 Wh kg^−1^ at a high power density of 2853 W kg^−1^, which is comparable to the previously reported organic cathodes for RMBs (Table S1).^[25]^ Notably, the electrode could still deliver a specific energy density of 51.25 Wh kg^−1^ and specific power of 8990 W kg^−1^ with a CE of 99.2 % even at a high current of 10 A g^−1^. Besides, the CuDEPP displays good cycling stability: the capacity retention is above 76 % after 500 cycles at 4 A g^−1^ with an average CE of 98 % and could retain a capacity of as high as 72 mAh g^−1^ (Figure 2c). Such stabilized capacity at high rates is outstanding among the previously reported organic and inorganic cathodes reported for RMBs (Figure 2d). It corresponds to an energy density of >92 Wh kg^−1^ at high power of >5100 W kg^−1^ (Figure S5).

A detailed kinetic analysis (Figure S6) was conducted to verify the fast kinetics and quantify the contribution of the surface‐controlled and diffusion‐controlled processes according to Equation (S1). Both diffusion‐ and surface‐controlled processes have significant contributions at low scan rates (Figure S6c). However, the contribution of the surface‐controlled current significantly increases with rising the sweep rate (Figure S6e). Typically, the estimated surface capacitive contribution is as high as 83 % at a scan rate of 10 mV s^−1^ (Figure S6d), indicating effective surface charge storage for the CuDEPP electrode. On the other hand, the diffusion‐controlled contribution becomes smaller upon increasing the scan rate, which could be considered as the reason for reduced total capacity at high scan rates. This decline is expected due to less time for double‐charged cations and bulky anions to diffuse through the bulk of the electrode material. Remarkably, the CV curves exhibit a nearly rectangular shape at a high scan rate of 100 mV s^−1^ (Figure S7), reflecting the rapid electron and ion transport as well as superb rate capability desired for high power energy storage.

Further evaluations confirmed that the capacity significantly increases with increasing the electrolyte concentration. The discharge capacity increases to 155 mAh g^−1^ after 20 cycles at 1 A g^−1^ using 0.45 m electrolyte; however, apparent capacity degradation was observed from 23 cycles (Figure 2e). We speculate that this decline might have primarily originated from the side reactions between the Mg anode and electrolyte rather than being due to the side reactions and/or degradation of CuDEPP cathode. As shown earlier by scanning electron microscopy (SEM) and energy‐dispersive X‐ray spectroscopy (EDS) analysis, the Mg metal soaked in the electrolyte is stable even after a week of immersion.^[11]^ However, as will be explained later, post‐mortem analysis of the cycled cells confirmed that side reactions occur between the Mg metal and electrolyte during the cycling. To clarify whether the instability of CuDEPP and/or side reactions contribute to the decline of the cell's capacity, the cycled cathode was reassembled in a new cell with a fresh Mg anode and renewed electrolyte and subjected to the cycling test. Surprisingly, the capacity was fully recovered to 155 mAh g^−1^ after a few cycles (Figure 2f), implying the impressive reversibility of the cathode in the Mg‐ion system even after 100 cycles. The almost overlapping cyclic voltammetry (CV) curves (Figure S8a) and the relatively stable voltage plateau and specific capacity in the reassembled cell (Figure S8b) further verify the outstanding stability of the Mg‐based energy storage system with the CuDEPP cathode.

To gain more insight into the electrochemical conditioning process in the initial cycles and the mutual influences on the CuDEPP and its energy storage mechanism, it is very useful to take a closer look at the CVs and the post‐mortem analysis of the cycled electrodes. A distinct oxidative peak was found in the first anodic scan at 3.1 V with respect to the reference electrode (Figure 3a). The intensity of this peak was significantly increased in the second anodic scan and subsequently reduced in the following scan. Clearly, no peak was observed from the fourth cycle onward. Similarly, a sharp irreversible reductive signal was found in the first cathodic scan at 0.55 V (Figure 3a).

**Figure 3:**
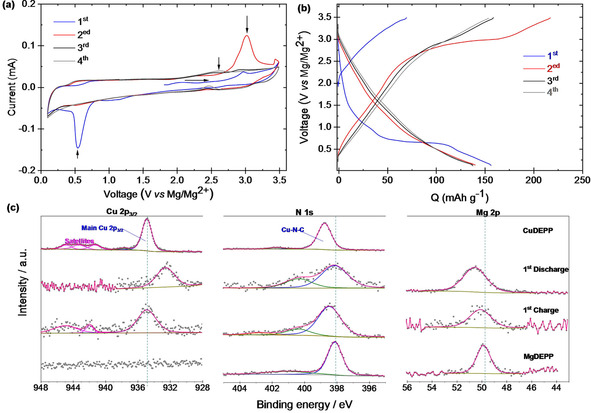
Electrochemical measurements of CuDEPP in the potential range of 0.1–3.5 V in the Mg[B(hfip)_4_]_2_ electrolyte. (a) CV curves of the CuDEPP electrode during the first four cycles scanned at 0.2 mV s^−1^. (b) Corresponding galvanostatic discharge‐charge profiles at 1 A g^−1^. (c) Ex‐situ XPS measurements of cathodes after the first discharge and first charge in comparison with the pristine CuDEPP and MgDEPP. Atomic ratios of N/Cu and N/Mg derived from corresponding XPS spectra are shown in Table S3.

The intensity of this peak was also significantly reduced in the subsequent cycles and gradually disappears. These results are also in accordance with the flat voltage plateaus at about 3.1 and 0.6 V (Figure 3b), which further confirm the irreversible changes of the active materials as a result of side reactions with the electrolyte. These suggest that there might be an electrochemical conditioning process that results in a stable cathode with reversible electrochemical reactions.

The irreversible oxidative peak at 3.1 V is most likely associated with the self‐conditioning feature of the CuDEPP as also observed in the Li‐ and K‐based systems.[^19^, ^26^] Surprisingly, the conditioning via polymerization of the ethynyl groups is not the case here as the characteristic infrared bands of the ≡C−H and −C≡C− do not disappear upon conditioning (Figure S9). To further evaluate the effect of the self‐conditioning of the CuDEPP, the electrolytes extracted from the cells charged or discharged to specific potentials were examined by UV/Vis spectroscopy. As depicted in Figure S10, Soret and Q‐bands of porphyrin have been detected in the extracted electrolytes and the intensity decreases in the charged cells. Interestingly, the bands are not detected in the fully charged state, which could be considered as a sign of the extremely reduced solubility upon in‐situ conditioning. This limited dissolution was further confirmed with the ex‐situ solubility testing of pristine and conditioned CuDEPP electrodes (Figure S11). Additional investigation of the cathodes indicated that the crystallinity was slightly reduced in the first discharge and further lowered in the successive charge process as shown by X‐ray diffraction (XRD) (Figure S12a). The reduced crystallinity can be ascribed to the strain/stress induced in the lattice of CuDEPP by large volume changes during insertion/deinsertion of bulky ions.^[27]^ On the other hand, the irreversible reductive peaks in the CV could be ascribed to the reduction of Cu^2+^ to Cu^+^. This phenomenon is also observed for CuDEPP in Mg(HMDS)_2_/MgCl_2_/PP_14_TFSI (Figure S13) and in KPF_6_ electrolytes.^[21]^ This attribution was confirmed by X‐ray photoelectron spectroscopy (XPS) as the Cu 2p peak in the first discharge state shifts approximately 2 eV to lower binding energies compared to the pristine material (Figure 3c). It is known to the authors that Cu^+^ and Cu^0^ have almost the same binding energy and their differentiation through Auger parameter was not feasible in this case because of low intensity of the Cu LMM peak. In agreement, the almost stoichiometric total N/Cu ratio in the CuDEPP (3.5) increases to 7.8 in the first discharge and further increases to 10.7 in the subsequent charge, and no Cu was detected upon further cycling (Table S3, Figure S14). The depletion of Cu from CuDEPP supports the reduction of Cu^2+^ to Cu^+^ and its transfer to the electrolyte due to the large ionic radius of Cu^+^.^[28]^ Indeed, a considerable amount of Cu is found on the Mg anode of first cycles at a binding energy around 932.5 eV (Figure S15). It is known in the literature that alongside the partial reduction to Cu^0^ and deposition on the Mg anode, the Cu^+^ can stably exist in the Mg[B(hfip)_4_]_2_ electrolyte and improve the reversibility of cathode reactions in the RMBs.^[29]^ However, we found no solid evidence that Cu^+^ can improve the reversibility of cathode reactions.

The Cu release from the active material can, in principle, lead to the formation of either (i) MgDEPP in the well‐known transmetalation reaction of the metalloporphyrins,^[30]^ or (ii) formation of the metal‐free porphyrin via demetallation reaction.^[28]^ UV/Vis spectroscopy (Figure S10) was used to shed light on this process. The spectrum of the CuDEPP displayed three absorption bands as a result of π→π* electron transition in the porphyrin core (one Soret and two Q bands), indicating D_4h_ symmetry for DEPP coordinated to Cu^2+^ through the four N‐heteronuclei. In the discharge, the spectral pattern changes from the two Q‐band spectrum to a four Q‐band spectrum, indicating D_2h_ symmetry of an intermediate, which is more soluble in the electrolyte. The number of absorption bands decreased in the charging above 2.3 V indicating the increased symmetry and probably a return to D_4h_ symmetry. Besides, a considerable red‐shift of the Soret band in both charge and discharge conditions (Figure S10b) agrees well with the predicted band gap reduction due to the Cu^2+^ substitution with Mg^2+^ in the DFT calculations (Figure S16).

Considering the above‐mentioned findings, intermediate participation of metal‐free porphyrin or porphyrin with both metal atoms in the course of the reaction is likely. The insertion of Mg into the porphyrin is possibly accompanied/followed by a transfer of the Cu^+^ atoms to the electrolyte. Although the affinity of Mg‐porphyrins to bind to two axial ligands^[31]^ may result in more complexity, the shift of N1s signal to lower binding energies in the XPS spectrum is consistent with the reference spectrum for MgDEPP (Figure 3c). The slight raise of N1s binding energy at the first charged state is likely due to the oxidation of MgDEPP. Furthermore, the abundance Mg ions in the first discharged state reduces to the almost stoichiometric N/Mg atomic ratio (Table S3) which further supports the formation of MgDEPP. Based on the discussed pieces of evidence, the following mechanism is suggested for the reductive transmetallation of the CuDEPP [Eqs. (1) and 2]:

(1) $${Cu}^{II}\left(DEPP\right)+\;e^{-}\to \;{Cu}^{II}{\left(DEPP\right)}^{-}$$

(2) $${Cu}^{II}{\left(DEPP\right)}^{-}\;\left(↔{Cu}^{I}DEPP\right)+\;{Mg}^{II}\to \;{Mg}^{II}{\left(DEPP\right)}^{-}+\;{Cu}^{I}$$

The higher tendency of the formed Mg‐porphyrin toward self‐association with axial ligands could be also a reason for retaining the crystallinity upon extended cycles (Figure S12b) and observing the self‐conditioning peak in the successive charging (Figure 2a).

The reversible anion/cation exchange was verified by XPS and XRD of the cycled cathode. In XPS, the B/Mg atomic ratio after 100 cycles is above 2 in the charged state, which confirms the [B(hfip)_4_]^−^ abundancy in the charging process. The ratio significantly reduces (to below 1) upon discharging, which suggests the withdrawal of the anion in the discharging process. The presence of B in the fully discharged electrode possibly indicates a partial insertion of [MgB(hfip)4]^+^,^[32]^ at least on the surface region. On the other hand, the N/Mg atomic ratio after 100 cycles decreases from around 1.5 in the charged state to almost 1 upon discharging, which suggests the introduction of the Mg ions in the discharging process. Besides, the XRD patterns of the cycled cathode (Figure S12b) indicate a gradual shift of the diffraction peaks at 2*θ* of 10.7 and 15.7° to a lower diffraction angle suggesting an expansion of the interplanar spacing as a result of the ion insertion.

To understand the capacity degradation despite the stability of the cathode (Figure 2f), ex‐situ SEM observation, EDS, and XPS analysis of the cycled anode from Mg−CuDEPP cells was conducted. Indeed, the Mg anode surface had turned black, rough and porous after 100 cycles (Figure S17) and EDS detected a significant mass of C, O, and F (Figure S18). The porous structure could be ascribed to a gradual consumption of a fraction of the Mg metal in the electrolyte solution due to the formation of MgDEPP and incomplete reversibility of Mg dissolution/deposition (≈98 %).^[10]^ On the other hand, detection of B, C, O, and F elements on the cycled anode in the EDS (Figure S18) and XPS (Figure S19) imply the decomposition of [B(hfip)_4_] anions on the Mg metal surface. This is consistent with the conclusion of the recent report utilizing Mg[B(hfip)_4_]_2_ in Mg/Cu asymmetric cells that the instability of the formed solid electrolyte interphase (SEI) on the Mg metal gradually blocks Mg‐ion transport, causing the poor long‐term electrochemical stability.^[33]^ In fact, significant advances in the formation of stable magnesium‐ion‐conducting surface films on the Mg anode surface (e. g., by using appropriate solvents, salts, and/or additives) are necessary to utilize the full potential of metalloporphyrins for RMBs.

In conclusion, we have revealed for the first time that reversible insertion/deinsertion of Mg^2+^ and bulky anions into metalloporphyrin can occur electrochemically and stably. We show that with an optimized electrolyte composition, CuDEPP can perform reversible two‐ or four‐electron redox reactions. The porous nature and facile reversible aromaticity switch in the cathode overcome the perceived sluggish kinetics of interaction with Mg ions and result in high rate performance and power density. Diverse ex‐situ analyses have been applied to elucidate the distinct CuDEPP behavior during cycling, providing an in‐depth understanding of the self‐conditioning and storage mechanisms. Despite the metal exchange and self‐conditioning in the initial cycles, the conditioned cathode is stable during charge/discharge cycling in different electrolytes, which is among the important requirements for considering further practical applications. Even the set‐up with two‐electron reversible redox reactions demonstrate a comparable energy density and stability to that of benchmark organic cathode materials in RMBs. However, the right combination of the cathode and electrolyte is crucial to uncover the full potential of metalloporphyrins for Mg‐ion batteries. This clearly illustrates the potential of metalloporphyrins for Mg‐ion batteries, paves the way for the developments of new high potential cathode materials, and signifies the need for stable Mg‐ion conducting surface films on the Mg anode surface.

## Conflict of interest

The authors declare no conflict of interest.

## Supporting information

As a service to our authors and readers, this journal provides supporting information supplied by the authors. Such materials are peer reviewed and may be re‐organized for online delivery, but are not copy‐edited or typeset. Technical support issues arising from supporting information (other than missing files) should be addressed to the authors.

Supplementary
