# Supplementary material for: A Self‐Conditioned Metalloporphyrin as a Highly Stable Cathode for Fast Rechargeable Magnesium Batteries
Source: ChemSusChem. 2021 Mar 16;14(8):1840–6. doi: 10.1002/cssc.202100340 (PMC8251709; doi:10.1002/cssc.202100340)
Supplement: Supplementary file 1 — Supplementary [file CSSC-14-1840-s001.pdf]

ChemSusChem

Supporting Information

# A Self-Conditioned Metalloporphyrin as a Highly Stable Cathode for Fast Rechargeable Magnesium Batteries

Ebrahim Abouzari-Lotf,* Raheleh Azmi, Zhenyou Li, Shirin Shakouri, Zhi Chen, Zhirong Zhao-Karger, Svetlana Klyatskaya, Julia Maibach, Mario Ruben,* and Maximilian Fichtner*© 2021 The Authors. ChemSusChem published by Wiley-VCH GmbH. This is an open access article under the terms of the Creative Commons Attribution License, which permits use, distribution and reproduction in any medium, provided the original work is properly cited.

## Table of Contents

Experimental Procedures ........ 2

Supporting Figures and Tables ........ 3

References ........ 15

## Experimental Procedures

*Material Preparation:* CuDEPP was prepared from commercially available 5,15-Dibromo-10,20-diphenyl-21H,23H-porphyrin in a three-stage synthetic procedure resulting in substituting of bromide atom with acetylene group as described in our previous report (yield ~ 40%).[1] To enhance the porosity and reduce the size of the crystalline CuDEPP powder, ball-milling was applied for 30 minutes. Breaking of the large CuDEPP crystals (**Figure S2**) upon milling and subsequent opening of the closed pores and formation of new pores due to the disordered arrangement of the crystals enhance the porosity and reduce the size of the crystalline CuDEPP powder. The magnesium bis-hexamethydisilazide-magnesium chloride complex ($Mg(HMDS)_2$-$4MgCl_2$) was synthesized in THF following an earlier report.[2] Subsequently, 1-butyl-1-methylpiperidinium bis(trifluoromethylsulfonyl)imide ($PP_{14}$-TFSI) ionic liquid (IL) was added and stirred for 48 h to prepare a Mg-containing IL solution with a total $Mg^{2+}$ concentration of 0.625 M and 25 vol% of IL. The magnesium tetrakis(hexafluoroisopropyloxy)borate $Mg[B(hfip)_4]_2$ electrolyte with $Mg^{2+}$ concentration of 0.3 and 0.45 M was synthesized from $Mg(BH_4)_2$ and hexafluoro-2-propanol in DME according to our previous report.[3]

*Characterization:* The milled CuDEPP powder was mixed with a mixture of carbons and poly(vinylidene difluoride) (PVDF) in a mass ratio of 5:4:1. A mixture of carbons was composed of Super P carbon black, graphene nanoplatelets (GNP) and carbon nanofiber (CNF) with a mass ratio of 50:45:5. N-methyl-2-pyrrolidinone (NMP) was added and the mixture was grounded for 20 min followed by casting onto a SS current collector. Vacuum drying of casted slurry at 110 °C for 12 h resulted in the electrodes with a loading mass of 1.5 to 2.5 mg $cm^{-2}$. Electrochemical measurements were carried out in CR2032 coin cells, in which a porous borosilicate glass fibre sheet GF/C was used as a separator and polished Mg foil anode. Galvanostatic cycling and CVs were recorded on Arbin BT2000 and VMP-3 workstation, respectively. The gravimetric energy density ($E_g$) and power density (P) of the CuDEPP cathode were calculated using the following equation:

$$E_g\left(\frac{Wh}{Kg}\right) = V_{av}(V) \times C_{cat}\left(\frac{mAh}{g}\right) \qquad \text{(eq:1)}$$

$$P\left(\frac{W}{Kg}\right) = \frac{E_g}{t_{dis}} \qquad \text{(eq:2)}$$

where $V_{av}$ is the average discharge voltage, $C_{cat}$ is the specific capacity of cathode and $t_{dis}$ is a discharge time (h).
IR spectra were recorded on a Nicolet iS50 FT-IR. Absorption spectra were collected using a Cary 5000 UV-Vis/NIR spectrophotometer. SEM imaging and EDX analysis were conducted with ZEISS LEO 1530 equipped with an energy dispersive x-ray spectrometer of X-maxN from Oxford instruments. XRD measurements were recorded on a Philips XPERT-MPD with monochromatic CuKα1 radiation (λ = 0.154 nm) and a step size of 0.02°. X-ray photoelectron spectroscopy measurements were performed using a K-Alpha instrument (Thermo Fisher Scientific, East Grinstead, UK), applying a microfocused, monochromated Al Kα X-ray source with 400 µm spot size. The K-Alpha charge compensation system was used during analysis, using electrons of 8 eV energy and low-energy argon ions to prevent any localized charge build up. Data acquisition and processing using the Thermo Avantage software are described in Parry et al.[4] The spectra were fitted with one or more Voigt profiles. The binding energies of powder samples such as CuDEPP and MgDEPP are referenced to C 1s peak at 285 eV. The C 1s peak of the graphite in as-prepared and OCV cathodes at 284.4 eV is taken as referencing energy for these samples. For cycled electrodes, the C 1s peak of $–CF_3$ species of electrolyte salt at 292.6 eV is considered as a referencing point (illustrated in **Figure S12**). Binding energies are controlled by means of the well-known photoelectron peaks of metallic Cu, Ag, and Au. For intense peaks and/or peaks clearly evidenced by the peak shape, the binding energy uncertainty was around ±0.1 eV. In the case of weak peaks and no direct justification by the peak shape, the uncertainty was set to ±0.2 eV. The analyzer transmission function, Scofield sensitivity factors,[5] and effective attenuation lengths for photoelectrons were applied for quantification. Effective attenuation lengths were calculated using the standard TPP-2M formalism.[6]

*Computational:* The calculation was done with the Gaussian 16 programs. Geometry optimizations were performed in the gas phase using B3LYP functional of the DFT method. Hydrogen, carbon and nitrogen atoms were treated with the 6-31G** basis set while cupper and magnesium atoms were treated with the LANL2DZ basis set.

## Supporting Figures

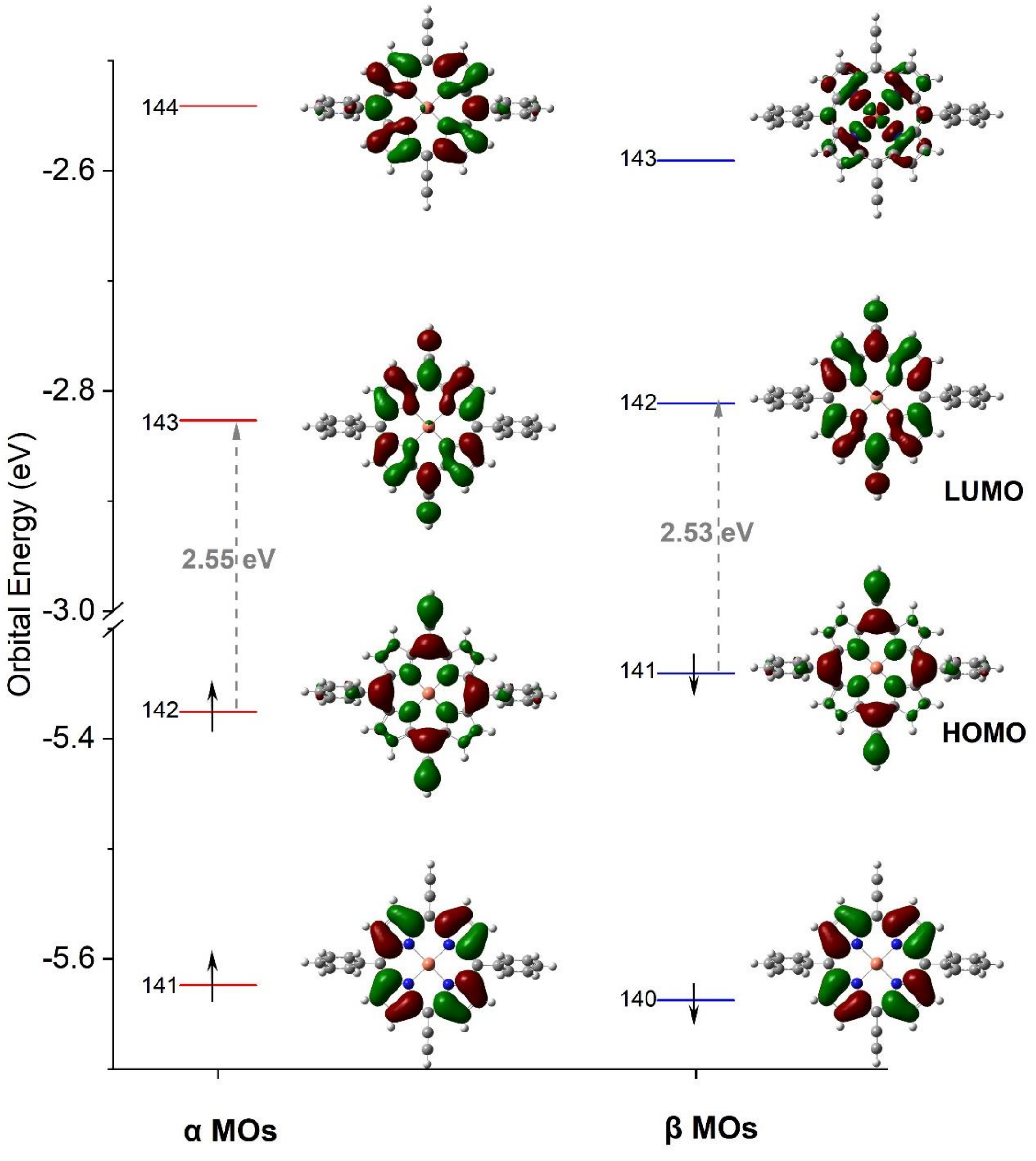


**Figure S1.** Energy level diagram of the frontier molecular orbitals of CuDEPP as estimated by DFT calculations. The gap is significantly lower than organic electrodes such as carbonyl compounds [7–10], Calix[6]quinone[11] and polyimides[12] as well as triazine - based materials.[13]

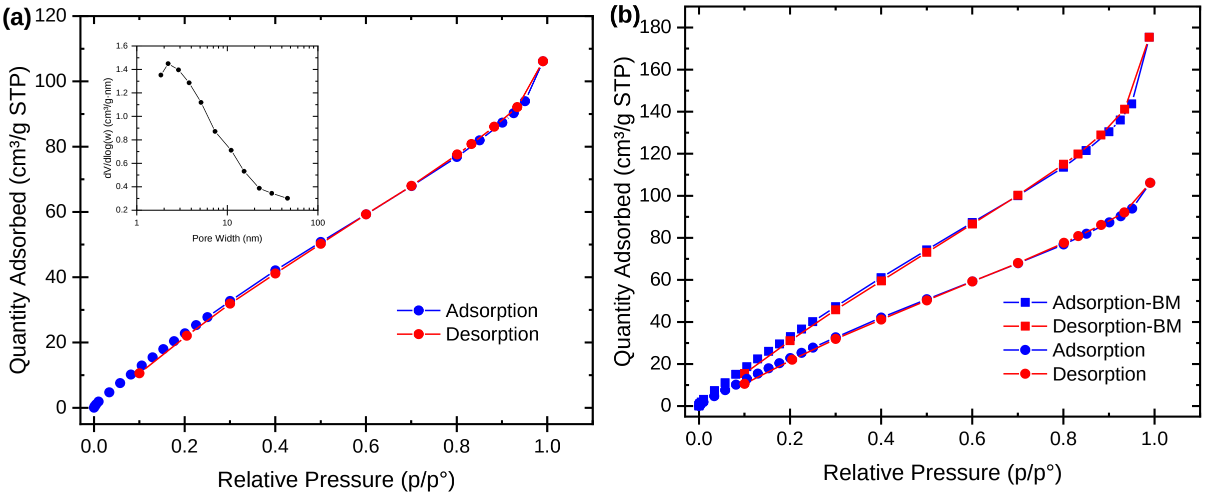


**Figure S2.** Nitrogen adsorption-desorption isotherms of (a) pristine and (b) ball-milled (BM) CuDEPP. The inset shows the pore size distributions.

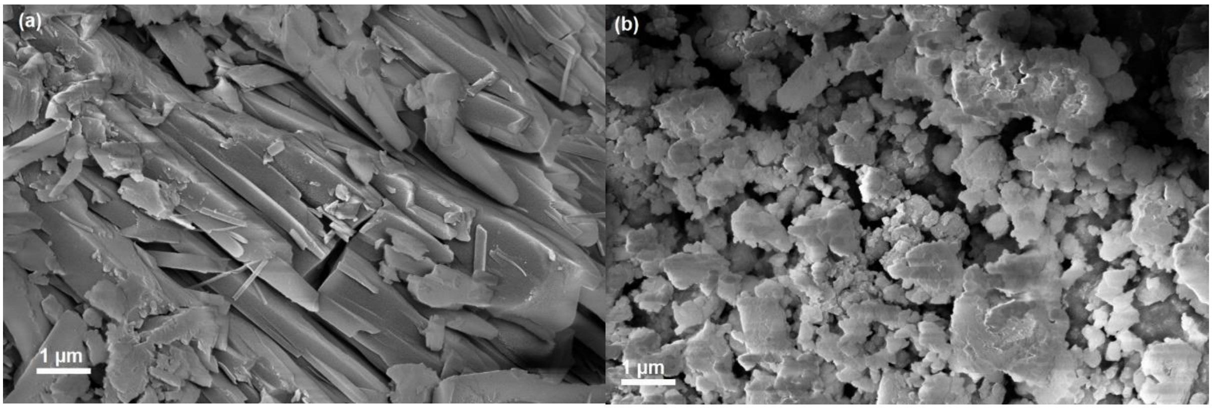


**Figure S3.** SEM micrographs of the (a) as-prepared and (b) milled CuDEPP.

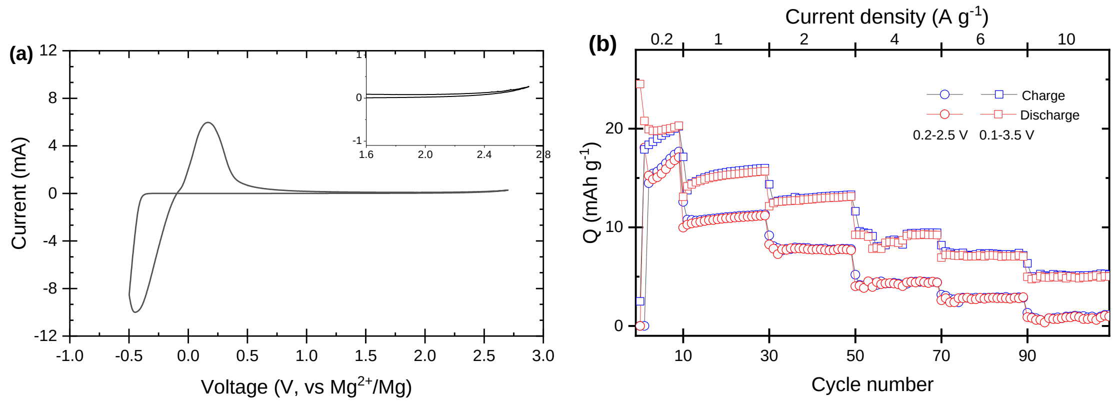


**Figure S4.** (a) Typical cyclic voltammograms of Mg deposition-stripping in 0.625 M $Mg^{2+}$ electrolyte of $Mg(HMDS)_2/MgCl_2/PP_{14}TFSI/THF$ on SS electrode at a scan rate of 20 mV $s^{-1}$. The insets show the enlargements of the voltage range of 1.6-2.8 V. (b) Cycling performance of inactive cathode components with $Mg(HMDS)_2/MgCl_2/PP_{14}TFSI/THF$ and $Mg[B(hfip)_4]_2$ electrolytes in the respective voltage range of 0.2-2.5 and 0.1-3.5 V *vs.* $Mg^{2+}/Mg$. .

Table S 1. An energy density comparison of Mg batteries with and without considering the mass of electrolyte.

| Cathode materials | Electrolyte | Energy density[a] (Wh/Kg) | Energy density[b] (Wh/Kg) | Remarks | Ref |
|---|---|---|---|---|---|
| P14AQ | $Mg(HMDS)_2$-$4MgCl_2$/2THF | 178 | 53 | | [14] |
| DMBQ | $Mg(TFSI)_2$-$2MgCl_2$/DME | - | 36 | | |
| COF | $Mg(TFSI)_2$/DME | 146.4 | - | | [15] |
| P(NDI2OD-T2) | | 74.9 | - | | |
| CuDEPP | $Mg(HMDS)_2$/$MgCl_2$/$PP_{14}$TFSI/THF | 132 | 55.1 | | This work |
| | | 74 | 36.0 | After 2000 cycles | |
| | $Mg[B(hfip)_4]_2$/DME | 186.7 | - | | |

[a] For the calculation of the performance parameters of cells utilizing CuDEPP without considering the mass of electrolyte, we used the following equation as described elsewhere:[15]

$$E_g\left(\frac{Wh}{Kg}\right) = V_{av}(V) \times C_{tot}\left(\frac{mAh}{g}\right) \qquad \text{(eq:3)}$$

$$C_{tot} = \frac{1}{C_{ca}^{-1}+C_{an}^{-1}+} \qquad \text{(eq:4)}$$

where $C_{ca}$ and $C_{an}$ are the specific capacity of cathode and anode, respectively.

[b] To calculate the energy density of Mg batteries by considering electrolyte mass, $C_{tot}$ was calculated according to equation 5:

$$C_{tot} = \frac{1}{C_{ca}^{-1}+C_{an}^{-1}+C_{el}^{-1}} \qquad \text{(eq:5)}$$

where $C_{el}$ is the apparent specific capacity of the electrolyte and calculated according to equation 6:

$$C_{el} = z \times F \times (M_W \times (c)^{-1})^{-1} \qquad \text{(eq:6)}$$

where z is the mole number of electrons transferred with the consumption of 1 mol of $MgCl_2$, $M_w$ is the molecular weight of $MgCl_2$, F is the Faraday constant, and c is the weight percentage of $MgCl_2$ in the electrolyte.

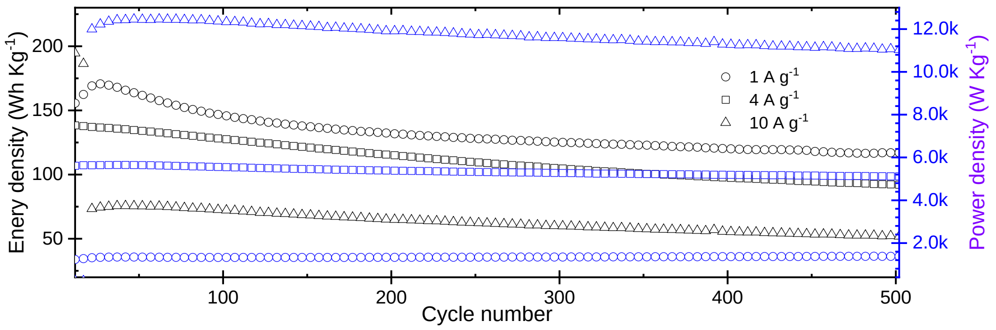


**Figure S5.** Long-term cycling performance of the CuDEPP in 0.3 M Mg[B(hfip)$_4$]$_2$ electrolyte in the voltage range of 0.1-3.5 V *vs.* $Mg^{2+}$/Mg.

**Table S2.** The capacity, average discharge voltage and cycling stability comparison of typical cathodes at various current densities for RMBs.

| Cathode materials | Electrolyte | Current density [mA $g^{-1}$] | Average Voltage (V) | Capacity[a] [mAh $g^{-1}$] | Cycling stability | Ref |
|---|---|---|---|---|---|---|
| 2-Ethylhexylamine pillared $VS_2$ nanoflowers | $Mg(HMDS)_2$-4$MgCl_2$/2THF-$PP_{14}$TFSI | 1000 | < 1 | 95 | 70% (600 cycles) | [16] |
| $Cu_3Se_2$ | BCM[b]/DME | 100 | < 1 | 250 | 80% (200 cycles) | [17] |
| $EVI_2$ [c] | $Mg(HMDS)_2$/TEGDME | 115 | 1.57 | 200 | 96% (100 cycles) | [18] |
| | | 1145 | 1.3 | 80 | 73% (500 cycles) | |
| $EVBr_2$ | | 1430 | 1.3 | 90 | 63% (500 cycles) | |
| Poly(hexaazatrinaphthalene) (PHATN) | not mentioned | 20 | 1.3 | 107 | ~90% (200 cycles) [d] | [19] |
| Active carbon cloth (ACC)/$I_2$ | $Mg(HMDS)_2$-$AlCl_3$-$MgCl_2$/TEGDME | 105.5 | ~ 2 | 180 | 94% (120 cycles) | [20] |
| $Mo_6S_8$ | $Mg[B(OCH(CF_3)_2)_4]^2$/DME | 45 | < 1 | 47 | 100% (400 cycles)[d] | [21] |
| Vat Orange 11 | APC | 500 | 1.5 | 57 | 67.0% (1000 cycles)[d] | [22] |
| $SnO_2$-rGO | APC | 100 | < 1 | 150 | 90.0% (150 cycles)[d] | [23] |
| | | 100 | < 1 | 120 | 98% (150 cycles)[d] | |
| $VS_4$ | APC | 50 | < 1 | 85 | 90% (400 cycles)[d] | [24] |
| $VS_4$-rGO | $Mg[B(hfip)_4]_2$/DME | 500 | 1.3 | 50 | 58% (350 cycles)[d] | [25] |
| 3,4,9,10-perylenetetracarboxylic dianhydride (PTCDA) | APC | 200 | 1.7 | 105 | 79% (200 cycles) | [26] |
| $Mo_{154}$[e] | APC | 500 | < 1 | 45 | 55% (500 cycles)[d] | [27] |
| Poly(antraquinoyl) sulfide (PAQS) | $MgCl_2$-$AlCl_3$/THF | 50 | ~1.4 | ~50 | 25% (100 cycles) | [28] |
| Poly(1,4-anthraquinone) P14AQ (P14AQ) | $Mg(TFSI)_2$-2$MgCl_2$/DME | 260 | ~1.4 | 140 | 67% (500 cycles) | [29] |
| | $MgCl_2$-$AlCl_3$/DME | 260 | ~1.6 | 95 | 35% (500 cycles)[d] | |
| | $Mg(HMDS)_2$-4$MgCl_2$/2THF | 520 | ~1.4 | 87 | 77 mAh $g^{-1}$ (1000 cycles)[h] | [30] |
| | $Mg(TFSI)_2$/ diglyme | 130 | 1.36 | 120 | 100% (100 cycles)[f] | [14] |
| P(NDI2OD-T2)[g] | | 300 | 1.4 | 50 | 87% (2500 cycles) | |
| COF | $Mg(TFSI)_2$/DME | 570 | 1.35 | 60 | 59% (300 cycles) | [15] |
| Polyimide/carbon nanotube PI/CNT | $Mg(HMDS)_2$-4$MgCl_2$/THF-$PP_{14}$TFSI | 1820 | ~1.4 | ~70 | 42 mAh $g^{-1}$ (8000 cycles)[h] | [12] |
| | | 3650 | ~1.4 | ~80 | 48 mAh $g^{-1}$ (8000 cycles)[h] | |
| **CuDEPP** | $Mg[B(hfip)_4]_2$/DME | 1000 | 1.4 | 111 | 66% (500 cycles) | |
| | | 4000 | 1.4 | 92 | 76% (500 cycles) | |

[a] After 100 cycles of charge-discharge and based on the weight of cathode active material

[b] Boron-centered anion-based Mg-ion electrolyte

[c] Ethyl Viologen

[d] Excluding initial condition cycle

[e] $Na_{15}\{[Mo_{154}O_{462}H_{14}(H_2O)_{70}]_{0.5}[Mo_{152}O_{457}H_{14}(H_2O)_{68}]_{0.5}\}$·ca. 400 $H_2O$ ($Mo_{154}$)

[f] Excluding first 10 cycles

[g] poly{[*N*,*N*′-bis(2-octyldodecyl)-1,4,5,8-naphthalenedicarboximide-2,6-diyl]-*alt*-5,5′-(2,2′-bithiophene)}

[h] The Cycling stability was not calculated in the source and data not clear to calculate.

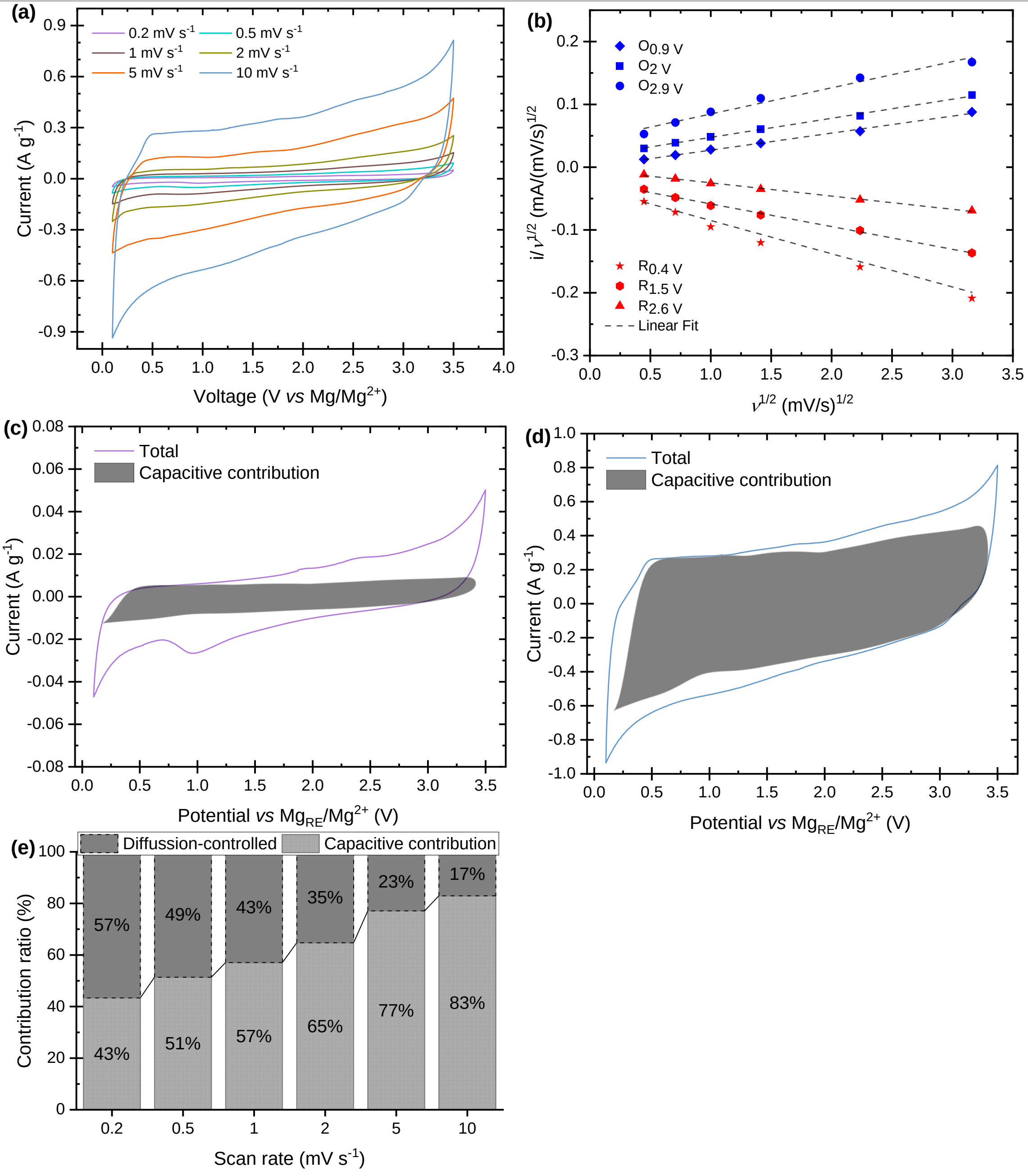


**Figure S6.** Electrochemical reaction kinetics of CuDEPP electrode in ($Mg[B(hfip)_4]_2$ electrolyte. (a) CV curves at various scan rates from 0.2 to 10 mV $s^{-1}$, (b) corresponding i(V)/$v$1/2 versus $v$1/2at various potentials during the charge-discharge process and (c) the calculated capacitive currents contribution (shaded regions) at 0.2 mV $s^{-1}$, and (d) at 10 mV $s^{-1}$. (e) Calculated contributions of diffusion and capacitive controlled charged storage at various scan rates.

The cyclic voltammetry data of the electrodes at different sweep rates (*v*) was analyzed according to [31]

$i_V = k_1 v + k_2 v^{1/2}$ **equation S1**

where the current response at a fixed potential ($i_V$) is derived from the surface capacitive ($k_1 v$) and diffusion-controlled ($k_2 v^{1/2}$) processes. The CV curves of CuDEPP electrode at various scan rates of 0.2-10 mV $s^{1/2}$ in Figure **S6a** was used to calculate $k_1$ and $k_2$ at each particular potential. **Figure S6b** shows a plot of the sweep rate dependence of the current according to eq S1 at selected oxidative and reductive potentials. The plots in the anodic and cathodic sweeps are almost linear (R-squared value of above 0.997) which enables us to determine $k_1$ and $k_2$ from the slope and the y-axis intercept, respectively. The CV curves exhibit a nearly rectangular shape at a high scan rate of 100 mV $s^{-1}$ (**Figure S7**), indicating excellent capacitive behavior and superb rate capability desired for high power energy storage.

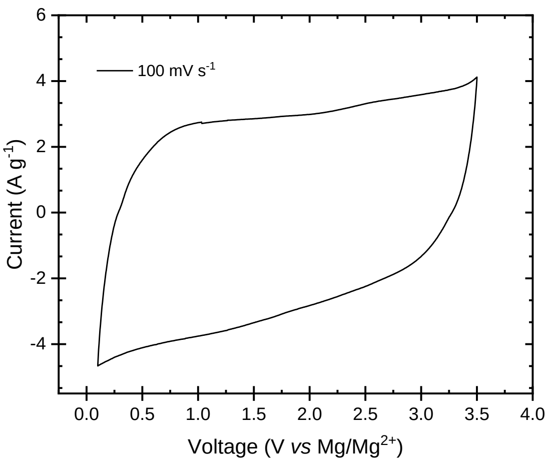


**Figure S7.** CV curves of CuDEPP at the scan rate of 100 mV $s^{-1}$.

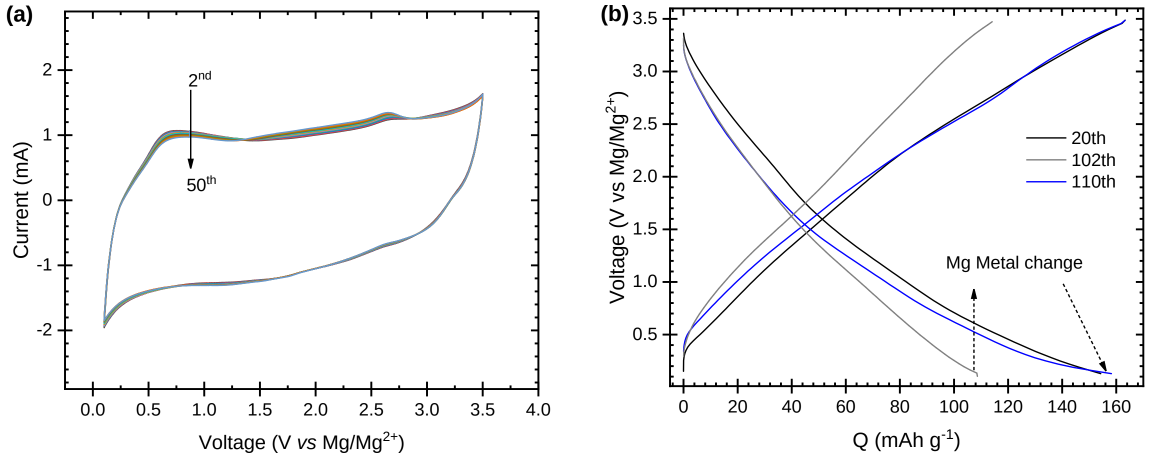


**Figure S8.** Electrochemical behaviour variation upon reassembling the cell after 100 cycles at 1 A $g^{-1}$ with a fresh Mg anode and renewed electrolyte: (a) CV curves at the scanning rate of 10 mV $s^{-1}$ and (b) charge-discharge profile at 1 A $g^{-1}$. CV curves of CuDEPP at the scan rate of 100 mV $s^{-1}$.

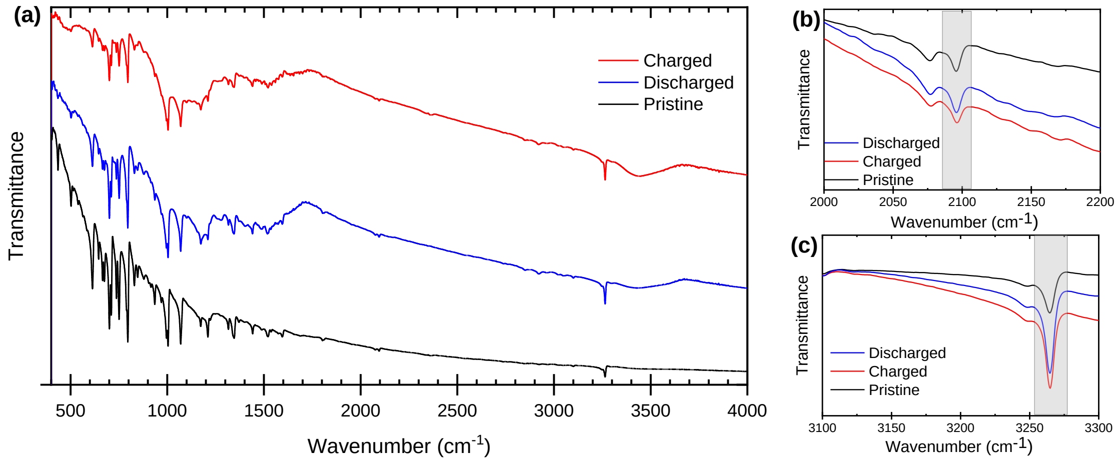


**Figure S9.** *Ex*-situ FT-IR spectra for pristine, 1st discharged, and 1st charged.

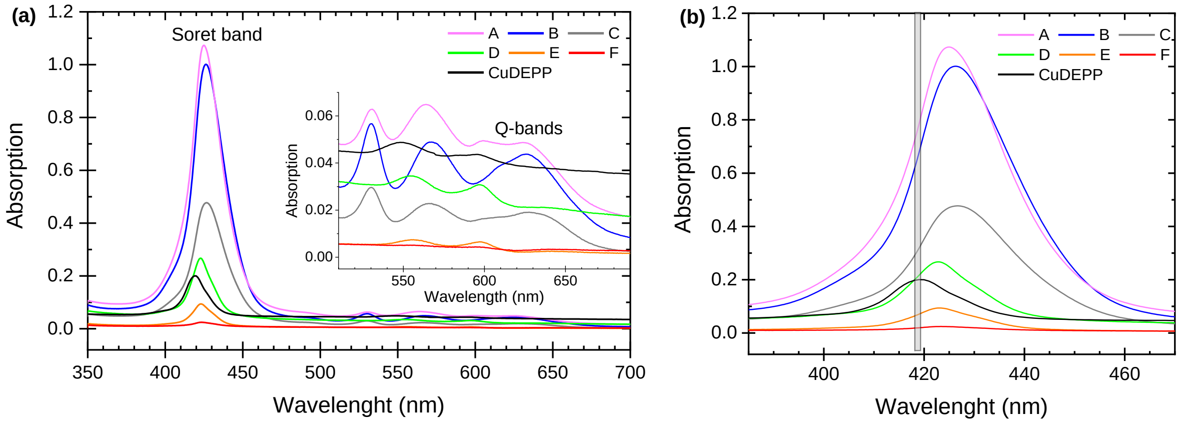


**Figure S10.** UV/Vis absorption spectra of extracted electrolytes from cells cycled to a specific potential of 0.6 V (A), 0.1 V (B), 2.3 V (C), 3 V (D), 3.1 V (E) and 3.5 V (F).

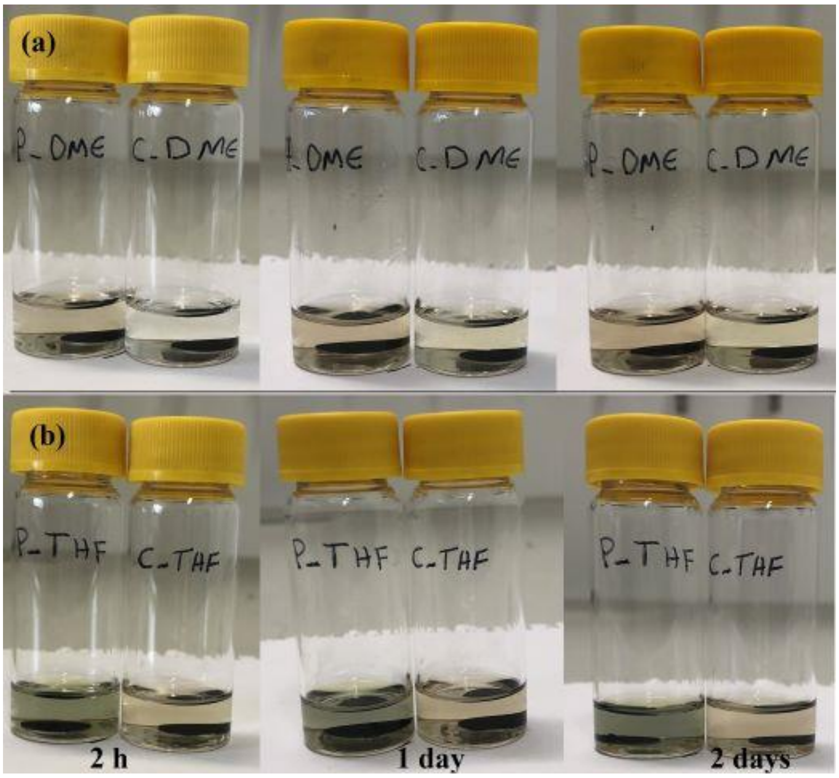


**Figure S11.** Pristine (P-) and conditioned (C-) CuDEPP electrodes immersed in (a) DME and (b) THF solvent for 2 h, 1day and 2 days. The limited solubility was observed for CuDEPP in DME and the solubility relatively increased in highly polar THF. However, the CuDEPP became considerably stable after the first cycle and very limited solubility was observed even in highly polar THF.

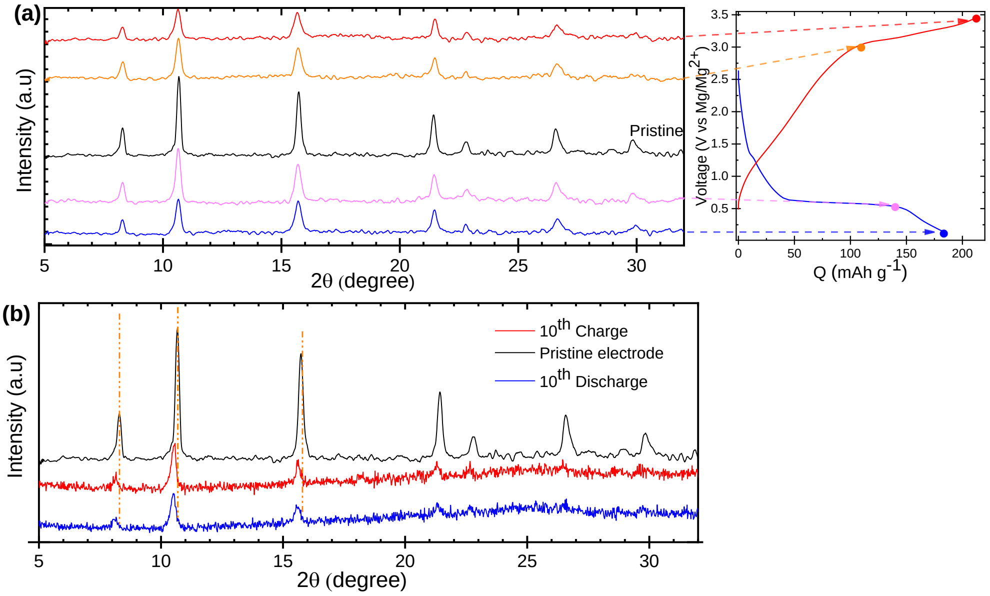


**Figure S12.** (a) The first discharge-charge curves and the corresponding *ex*-situ XRD patterns for CuDEPP in different potentials and (b) after 10th cycles.

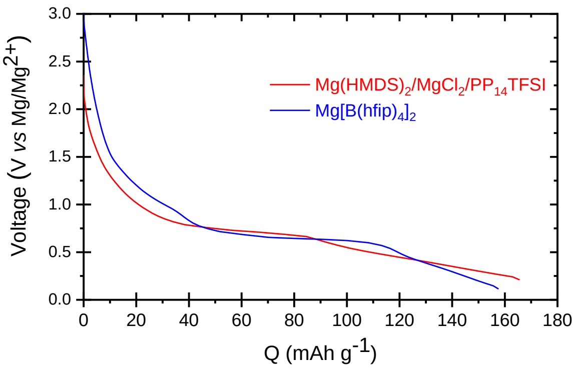


**Figure S13.** 1st Discharge curve with $Mg(HMDS)_2/MgCl_2/PP_{14}TFSI$ and $Mg[B(hfip)_4]_2$ electrolytes in the respective voltage range of 0.2-2.5 and 0.1-3.5 V *vs.* $Mg^{2+}/Mg$.

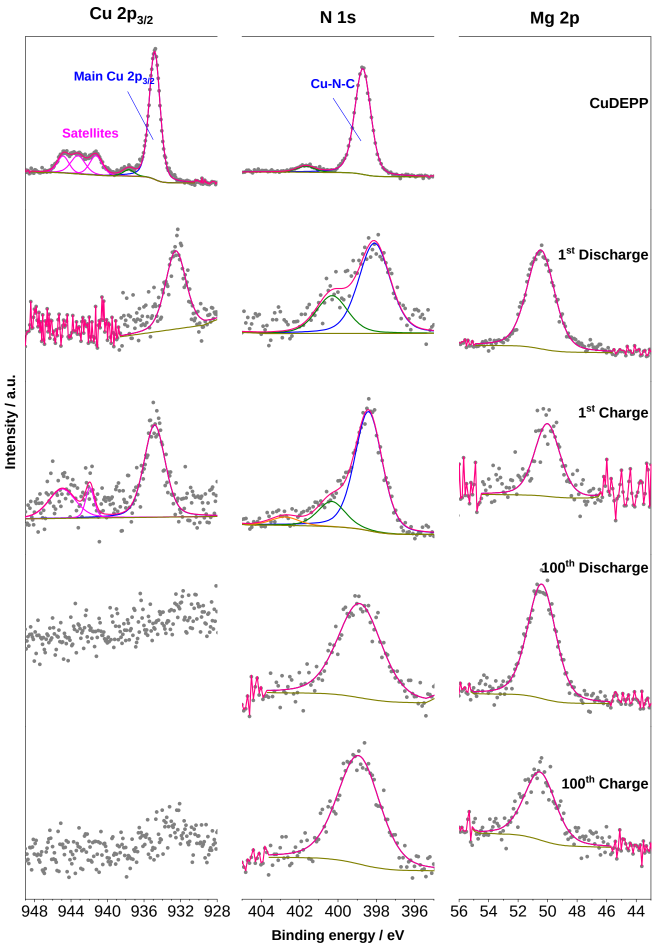


**Figure S14.** Cu $2p_{3/2}$, N 1s, and Mg 2p XP spectra of the pristine CuDEPP, and after $1^{st}$ and $100^{th}$ discharges and charges.

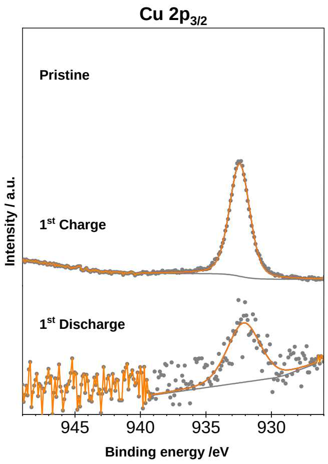


**Figure S15.** *Ex*-situ XPS Cu $2p_{3/2}$ spectra of pristine Mg anodes, after $1^{st}$ discharge and $1^{st}$ charge.

**Table S3.** N/Cu, N/Mg, and B/Mg ratio in the cathode active material and electrodes after cycling obtained by XPS.

| | CuDEPP | 1st discharge | 1st charge | 100th discharge | 100th charge | MgDEPP |
|---|---|---|---|---|---|---|
| N : Cu | 3.5 : 1 | 7.8 : 1 | 10.7 : 1 | No Cu | No Cu | - |
| N : Mg | - | 0.3 : 1 | 4.1 : 1 | 0.5 : 1 | 1.5 : 1 | 4 : 1 |
| B : Mg | - | 0.6 : 1 | 1.2 : 1 | 0.6 : 1 | 2.4 : 1 | - |

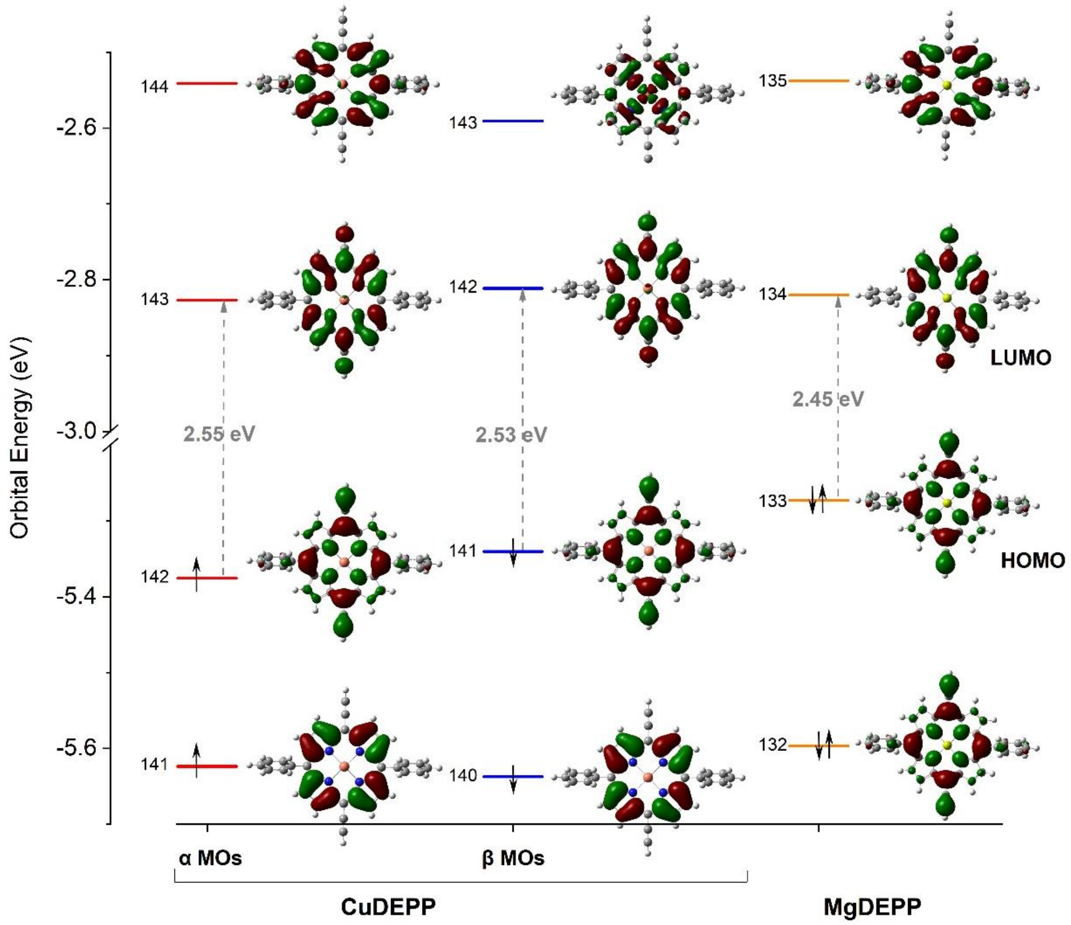


**Figure S16.** Molecular orbital and energies of CuDEPP and MgDEPP based on DFT-B3LYP calculations.

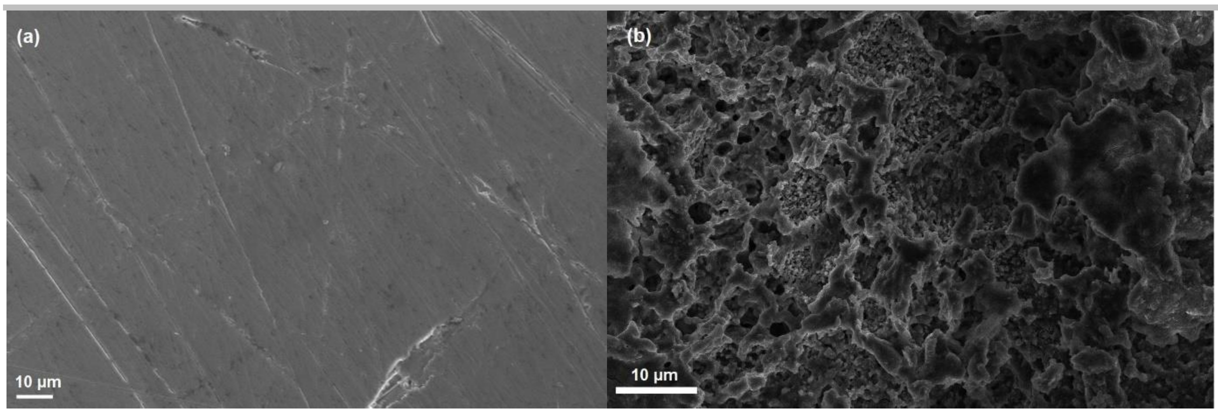


**Figure S17.** SEM images of (a) a pristine Mg anode and (b) a cycled Mg anode in $(Mg[B(hfip)_4]_2$ electrolyte revealing a rough and porous surface after 100 charge-discharge cycles at 1 A $g^{-1}$.

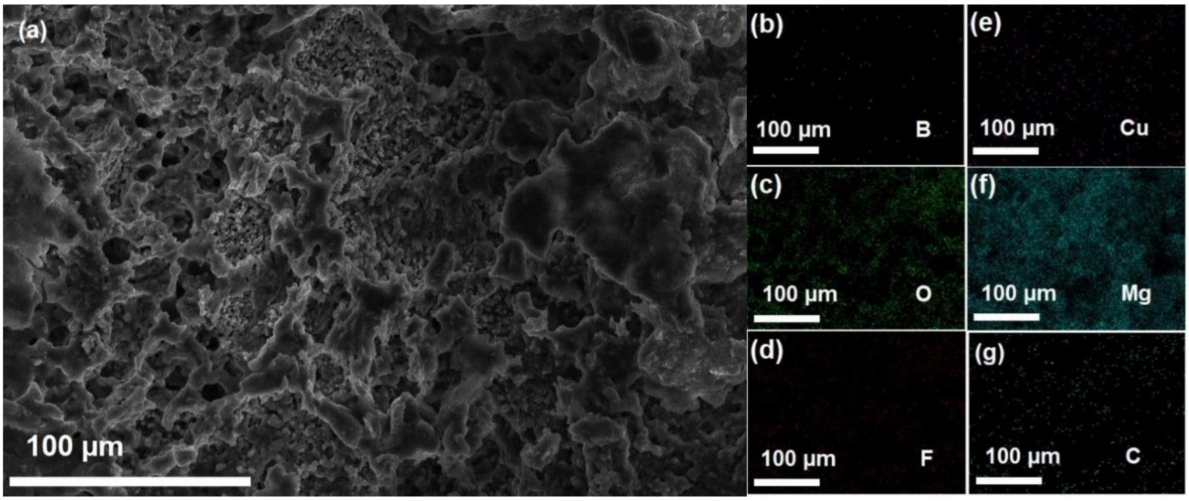


**Figure S18.** (a) SEM image of the Mg anode surface after 100 cycles at 1 mA $cm^{-2}$ (a) and corresponding elemental mapping of B, O, F, Cu, Mg and C (b-g, respectively).

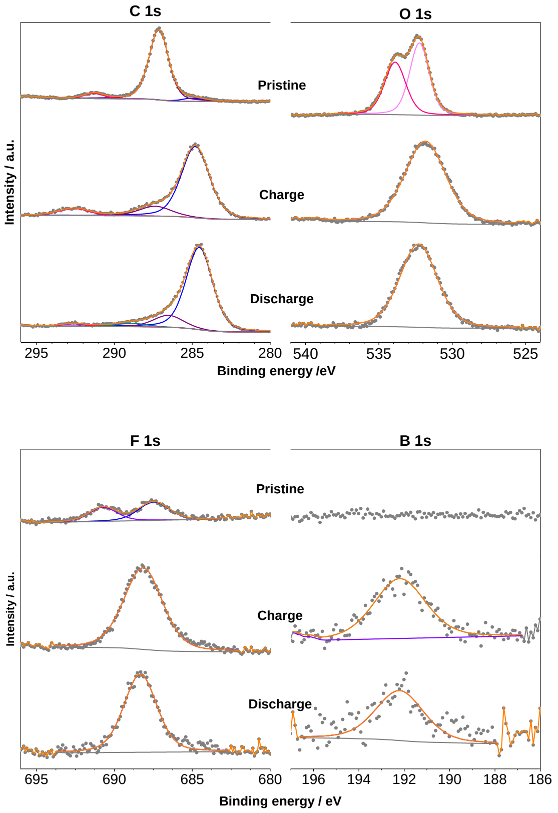


**Figure S19.** XPS C 1s, O 1s, F 1s, and B 1s spectra of pristine and cycled Mg anode.

## References


[1] P. Gao, Z. Chen, Z. Zhao-Karger, J. E. Mueller, C. Jung, S. Klyatskaya, T. Diemant, O. Fuhr, T. Jacob, R. J. Behm, et al., *Angew. Chemie Int. Ed.* **2017**, *56*, 10341–10346.
[2] C. Liao, N. Sa, B. Key, A. K. Burrell, L. Cheng, L. A. Curtiss, J. T. Vaughey, J.-J. Woo, L. Hu, B. Pan, et al., *J. Mater. Chem. A* **2015**, *3*, 6082–6087.
[3] Z. Zhao-Karger, M. E. Gil Bardaji, O. Fuhr, M. Fichtner, *J. Mater. Chem. A* **2017**, *5*, 10815–10820.
[4] K. L. Parry, A. G. Shard, R. D. Short, R. G. White, J. D. Whittle, A. Wright, *Surf. Interface Anal.* **2006**, *38*, 1497–1504.
[5] J. H. Scofield, *J. Electron Spectros. Relat. Phenomena* **1976**, *8*, 129–137.
[6] S. Tanuma, C. J. Powell, D. R. Penn, *Surf. Interface Anal.* **2011**, *43*, 689–713.
[7] Y. Ding, Y. Li, G. Yu, *Chem* **2016**, *1*, 790–801.
[8] K. Li, Q. Li, Y. Wang, H. Wang, Y. Li, Z. Si, *Mater. Chem. Front.* **2020**, DOI 10.1039/D0QM00335B.
[9] G. S. Jeong, K. H. Jung, S. Choi, K. C. Kim, *ACS Sustain. Chem. Eng.* **2020**, *8*, 11328–11336.
[10] K. H. Jung, G. S. Jeong, J. B. Joo, K. C. Kim, *ChemSusChem* **2019**, *12*, 4968–4975.
[11] W. Huang, S. Zheng, X. Zhang, W. Zhou, W. Xiong, J. Chen, *Energy Storage Mater.* **2020**, *26*, 465–471.
[12] Y. Wang, Z. Liu, C. Wang, Y. Hu, H. Lin, W. Kong, J. Ma, Z. Jin, *Energy Storage Mater.* **2020**, *26*, 494–502.
[13] Z. Lei, X. Chen, W. Sun, Y. Zhang, Y. Wang, *Adv. Energy Mater.* **2019**, *9*, 1801010.
[14] H. Dong, Y. Liang, O. Tutusaus, R. Mohtadi, Y. Zhang, F. Hao, Y. Yao, *Joule* **2019**, *3*, 782–793.
[15] R. Sun, S. Hou, C. Luo, X. Ji, L. Wang, L. Mai, C. Wang, *Nano Lett.* **2020**, *20*, 3880–3888.
[16] X. Xue, R. Chen, C. Yan, P. Zhao, Y. Hu, W. Kong, H. Lin, L. Wang, Z. Jin, *Adv. Energy Mater.* **2019**, *9*, 1900145.
[17] X. Cheng, Z. Zhang, Q. Kong, Q. Zhang, T. Wang, S. Dong, L. Gu, X. Wang, J. Ma, P. Han, et al., *Angew. Chemie Int. Ed.* **2020**, anie.202002177.
[18] Y. Sun, Q. Zou, Y. Lu, *Adv. Energy Mater.* **2019**, *9*, 1903002.
[19] M. Mao, C. Luo, T. P. Pollard, S. Hou, T. Gao, X. Fan, C. Cui, J. Yue, Y. Tong, G. Yang, et al., *Angew. Chemie Int. Ed.* **2019**, *58*, 17820–17826.
[20] H. Tian, T. Gao, X. Li, X. Wang, C. Luo, X. Fan, C. Yang, L. Suo, Z. Ma, W. Han, et al., *Nat. Commun.* **2017**, *8*, 14083.
[21] H. Xu, Z. Zhang, Z. Cui, A. Du, C. Lu, S. Dong, J. Ma, X. Zhou, G. Cui, *Electrochem. commun.* **2017**, *83*, 72–76.
[22] T. Debashis, H. M. Viswanatha, M. N. K. Harish, S. Sampath, *J. Electrochem. Soc.* **2020**, *167*, 070561.
[23] M. Asif, M. Rashad, J. H. Shah, S. D. A. Zaidi, *J. Colloid Interface Sci.* **2020**, *561*, 818–828.
[24] Z. Li, S. Ding, J. Yin, M. Zhang, C. Sun, A. Meng, *J. Power Sources* **2020**, *451*, 227815.
[25] Z. Li, B. P. Vinayan, P. Jankowski, C. Njel, A. Roy, T. Vegge, J. Maibach, J. M. G. Lastra, M. Fichtner, Z. Zhao-Karger, *Angew. Chemie Int. Ed.* **2020**, *59*, 11483–11490.
[26] L. Cui, L. Zhou, K. Zhang, F. Xiong, S. Tan, M. Li, Q. An, Y.-M. Kang, L. Mai, *Nano Energy* **2019**, *65*, 103902.
[27] X. Fan, S. Garai, R. R. Gaddam, P. V. Menezes, D. P. Dubal, Y. Yamauchi, P. W. Menezes, A. K. Nanjundan, X. S. Zhao, *Mater. Today Chem.* **2020**, *16*, 100221.
[28] J. Bitenc, K. Pirnat, T. Bančič, M. Gaberšček, B. Genorio, A. Randon-Vitanova, R. Dominko, *ChemSusChem* **2015**, *8*, 4128–4132.
[29] J. Bitenc, K. Pirnat, E. Žagar, A. Randon-Vitanova, R. Dominko, *J. Power Sources* **2019**, *430*, 90–94.
[30] B. Pan, J. Huang, Z. Feng, L. Zeng, M. He, L. Zhang, J. T. Vaughey, M. J. Bedzyk, P. Fenter, Z. Zhang, et al., *Adv. Energy Mater.* **2016**, *6*, 1600140.
[31] M. Forghani, S. W. Donne, *J. Electrochem. Soc.* **2018**, *165*, A664–A673.
